# Impact of sensory training on healthcare provider care for neurodivergent children

**DOI:** 10.3389/fped.2026.1840054

**Published:** 2026-07-17

**Authors:** Michele Kong, Jeremiah A. Tella, Chelsea Brown, Meg Raby, Deena Elsheikh, Brittney Johnston, Jennifer Conway, Uma Srivastava, Julian Maha, Inmaculada Aban

**Affiliations:** 1 Department of Pediatrics, University of Alabama, Birmingham, AL, United States; 2 Children's of Alabama, Birmingham, AL, United States; 3 KultureCity, Birmingham, AL, United States

**Keywords:** children, healthcare providers, hospital, neurodivergence, sensory training, neurodivergent

## Abstract

**Importance:**

Neurodivergent pediatric patients often experience sensory and communication challenges during hospitalization, yet formal provider training remains limited.

**Objective:**

To determine whether a structured sensory training can increase healthcare provider knowledge and perceived care for neurodivergent children.

**Design:**

A sensory training was implemented at a tertiary children's hospital to educate providers on the care of neurodivergent patients. Anonymous unmatched surveys that quantified commonality, knowledge, and comfortability via scaled responses of 1–10 as well as an 11-question sensory knowledge quiz were administered before and after implementation of the training. Survey responses represented aggregate cross-sectional samples collected at different time points.

**Setting:**

Single center tertiary children's hospital from 2021 to 2025.

**Participants:**

Medical staff including registered nurses, Certified Registered Nurse Anesthetists (CRNA), technicians, clinical assistants, and physicians.

**Main outcome and measure:**

Provider-reported knowledge, comfort, and perceptions related to sensory-informed care measured through survey and quiz responses.

**Results:**

Sensory training was administered to 1639 participants who were aged 21–30 years (67.91%), Caucasian (76.45%), female (92.80%), registered nurses (73.70%), and with <5 years of experience (77.36%). Pre-training commonality, knowledge, and comfort varied by age, job type, and prior neurodivergent experience. Younger respondents and those with fewer years of experience reported higher commonality. Knowledge and comfort increased with age, varied with job description, and greater among those with prior neurodivergent experience. Pre-training mean (±SD) scores for perceived commonality, knowledge, and comfort were 8.25 ± 1.88, 5.14 ± 2.07, and 6.09 ± 2.27, respectively. Higher mean post-training scores were observed across all domains compared with pre-training survey responses (9.09 ± 1.35, 7.81 ± 1.45, and 7.90 ± 1.55). Higher sensory knowledge quiz scores were observed in post-training assessments compared with pre-training assessments (pre: 8.42 ± 1.53 vs post: 9.42 ± 1.53). At follow-up assessment conducted beyond 12 months after initial training, mean scores remained higher than baseline pre-training responses across all measured domains (*n* = 453, 8.82 ± 1.50 for commonality, 7.02 ± 1.61 for knowledge, and 7.61 ± 1.73 for comfort). Sensory tools were rated as the most beneficial intervention (86.43%) followed by staff training (79.12%).

**Conclusions and relevance:**

Higher scores were observed in provider-reported knowledge, comfort, and perceived preparedness following implementation of the sensory training, with similar trends present at follow-up assessment.

## Highlights

**Question:** Can a hospital-based sensory training initiative support provider-reported preparedness related to caring for neurodivergent pediatric patients?
**Findings:** In this survey-based study including 1,639 individuals, higher scores were seen in provider-reported knowledge, comfort, and perceived preparedness related to caring for neurodivergent children with sensory processing differences following implementation of a sensory training.
**Meaning:** These findings support the feasibility and perceived educational value of sensory-focused training initiatives within a pediatric hospital setting.

## Introduction

The hospital with its unfamiliar and unpredictable environment, medical equipment, and alarms can be an overwhelming place for children who are *neurodivergent* or those with sensory processing difficulties, especially when they are feeling unwell. “Neurodivergent” is a nonmedical term referring to differences in how certain individuals process information and sensory input (1). Sensory processing terms describe how the body receives and interprets information from all senses. For example, interoception (internal body signals like hunger or heart rate), proprioception (body position and movement), vestibular (balance and spatial orientation), as well as the five traditional senses of vision (sight), audition (hearing), olfaction (smell), gustation (taste), and tactile (touch). Sensory processing challenges are atypical responses to stimuli, manifesting as either hypersensitivity to hyposensitivity to environmental inputs (2). While sensory processing challenges are often seen in children with autism spectrum disorder (ASD), attention deficit hyperactivity disorder (ADHD), prematurity, fetal alcohol syndrome, trisomy 21, and fragile X syndrome, they can occur with any comorbid condition or may exist in the absence of any diagnosed comorbid condition (3, 4).

Within the hospital, children are exposed to many adverse stimuli, including bright lights, loud or unfamiliar sounds, novel scents, and tactile sensations. They also sustain frequent interactions with unfamiliar healthcare providers and staff members. Hospital admission commonly disrupts a child's routine and is often accompanied by high medical acuity. Families may have little time to prepare for hospitalization and medical procedures. For children with social, communication, or sensory differences, these factors can lead to stress exacerbation and potential delays in care.

We recently developed a novel clinical pathway, designed to improve care for neurodivergent patients with sensory barriers by equipping staff with on-time training and sensory toolkits, and by integrating families early in the hospital course (4). In a follow up patient and family survey, improved hospital experience for these patients and their families was demonstrated, particularly with the immediate availability of tools to help with sensory regulation and improved overall staff encounters (5). In this quality improvement (QI) initiative, we evaluated provider-reported knowledge, comfort, and perceptions related to caring for neurodivergent patients before and after a sensory training designed for medical providers to support sensory-informed care practices.

## Methods

### Overall design

In partnership with KultureCity (http://www.kulturecity.org), a nonprofit organization with a broad network of neurodivergent individuals and families of children with sensory processing differences, caregivers were engaged to guide the development of the sensory training. Their input identified key barriers in hospital care and resources to improve sensory-inclusive practices. These insights, together with existing evidence, informed the creation of an implementation-ready training program developed by an interprofessional team of physicians, nurses, occupational therapists, applied behavior analysts, speech therapists, and child life specialists. The training course was designed to enhance healthcare providers’ understanding and management of sensory processing challenges in patients admitted to the hospital with medical diagnoses; focused on understanding sensory processing disorders, adapting methods of engagement and communications, implementing safeguards against potential sensory overloads, and familiarizing with available sensory tools and resources.

Medical staff including registered nurses (RN), Certified Registered Nurse Anesthetists (CRNA), patient care technicians, clinical assistants, and physicians with varying levels of experience participated in the sensory training. This project was conducted as a hospital-wide QI initiative using anonymous unmatched surveys collected at multiple time points to evaluate provider perceptions and knowledge related to sensory-informed care. A pre-training survey assessment was conducted to ascertain staff perception of how common, how knowledgeable and how comfortable they felt in taking care of neurodivergent patients. Responses were measured using a 10-point Likert scale (1- not at all knowledgeable/comfortable; 10- extremely knowledgeable/comfortable). Baseline demographic data (age, gender, self-identified ethnicity, job title, years of work experience, prior experience with neurodivergent patients) was collected and correlated with the pre-survey assessments. The sensory training was delivered through multiple live 60-minute sessions that was conducted across hospital units and shifts to maximize staff accessibility and participation. Participants attended one single first session. Although individual attendance was not linked to survey responses, surveys were distributed only to individuals physically present during the training sessions.

Following completion of the course, an identical survey was administered using the same scale. A knowledge assessment, consisting of an 11-question quiz related to the sensory training curriculum, was also conducted prior to and after the training course. A refresher training course was given at 12–24 months following initial training to reinforce key principles. Prior to the refresher training, participants completed an identical survey to assess retention. Participants were also asked to identify which components of the course (training, patient assessment, or sensory tools) they perceived as most beneficial in improving engagement with neurodivergent patients.

### Statistic

Summary statistics for the demographic and background characteristics of the responses were presented as means and standard deviations (SDs) for continuous variables, and as counts and percentages for categorical variables. Boxplots and bar charts were created to visualize the distribution of the scores and to compare them across periods or groups. The three areas of interest were: (1) how common participants perceived sensory barriers to be, (2) how knowledgeable participants perceived themselves to be about patients with sensory barriers, and (3) how comfortable participants perceived themselves to be in taking care of pediatric patients with sensory barriers. The pre- and post-training surveys were de-identified and, therefore, unmatched with no linking identifiers utilized. Analysis of variance (ANOVA) was used to assess differences in pre-training responses across demographic groups at a 5% significance level. Pairwise comparisons were conducted only for outcome–demographic variable combinations with a significant overall *F*-test (p-value < 0.05). For these comparisons, unadjusted p-values, mean differences, and standard errors were reported. Statistical significance for pairwise comparisons was determined by comparing the unadjusted p-values against Bonferroni-adjusted thresholds based on the number of comparisons. The corresponding significance cutoffs were: *p* < 0.008 (0.05/6) for age, *p* < 0.0005 (0.05/10) for ethnicity, *p* < 0.0003 (0.05/15) for employment title, and *p* < 0.008 (0.05/6) for years of experience. Ninety-five percent confidence intervals were constructed to estimate and compare mean scores across the three time points. All analyses were conducted using SAS version 9.4.

Power Consideration: As this was a QI initiative, there were no pre-study power calculation. The sample size simply reflects the available respondents over the study period who participated in the training.

### Ethics

The study was deemed by the University of Alabama at Birmingham's Institutional Review Board (IRB-300013122) to be exempt from review and qualified as a QI project to systematically educate and assess existing pre- and post-training knowledge of sensory processing challenges, sensory overload and regulation in pediatric patients. Because it was a QI project, no informed consent was obtained from the participants. Additionally, there was no initial control group. Completion of surveys and quiz was voluntary, and all data collected was deidentified, without linking. Participant confidentiality was maintained by using fully anonymous, paper-based surveys with no collection of identifiers at any stage. Prior to the training, participants completed a pre-survey and deposited it into a designated collection box; following the session, a post-survey was completed and placed into a separate collection box. No names, or other identifying information were recorded, and surveys were not linked at the individual level. All responses were analyzed in aggregate, ensuring that no individual participant could be identified. Guidelines for reporting QI initiatives published by the SQUIRE Development Group were consulted for this manuscript (6).

## Results

### Participant demographic

A total of 1,639 diverse individuals participated in the pre-training survey. Most respondents were between 21 and 30 years old (67.9%), female (92.8%), and self-identified as Caucasian (76.5%). The majority held the title of registered nurse (73.7%) and had 0–5 years of experience (77.4%). Of respondents, 74.1% reported having prior experience working with neurodivergent children. Table 1 summarizes participant demographic information.

**Table 1:** Participant demographics.

Characteristic	Frequency	Percentage
Age		
21–30	1113	67.91
31–40	270	16.47
41–50	120	7.32
Over 50	134	8.18
Unanswered	2	0.12
Gender		
Female	1521	92.80
Male	95	5.80
Other	1	0.06
Unanswered	22	1.34
Ethnicity		
Caucasian	1253	76.45
African American	289	17.63
Asian	31	1.89
Hispanic	33	2.01
Other	20	1.83
Unanswered	3	0.18
Title		
RN	1208	73.70
CRNA	16	0.98
Physicians	28	1.71
Technician	45	2.75
PCT/CA	47	2.87
Other	285	17.39
Unanswered	10	0.61
Experience		
0–5 years	1268	77.36
5–10 years	162	9.88
10–20 years	110	6.71
Over 20 years	91	5.55
Unanswered	8	0.49
Experience with Neurodivergent Patients		
Yes	1213	74.05
No	385	23.50
Unanswered	40	2.44

RN, registered nurse; CRNA, Certified Registered Nurse Anesthetists; MD, Medical Doctor; PCT, patient care technician; CA, clinical assistant.

### Pre-training survey scores varied based on demographic variables

Observed mean scores (95% CIs) for pre-training survey items were summarized by demographic characteristics (Supplementary Table 1). ANOVA results comparing mean scores for perceived commonality, knowledge, and comfort across demographic groups are presented in Table 2. Perceived commonality varied by age and experience: participants aged 21–30 reported higher scores than those aged 41–50 and >50, and those with 0–5 years of experience scored higher than those with 10–20 years. Females and individuals without prior experience caring for neurodivergent patients also reported higher scores. Knowledge differed by age, job type, and prior experience. Participants aged 41–50 had higher scores than 21–30, and those with prior neurodivergent care experience had higher scores than those without. Nurses (RN) had higher scores than physicians, while physicians and technicians had lower scores than the other category (any other training participant that did not fit into all other pre-defined job descriptions such as RN, CRNA, MD, technicians and CA). Comfort similarly varied by age, job type, and prior experience. Participants aged 31–40 and 41–50 had higher scores than 21–30. Physicians reported lower scores than all the job types, and those with prior experience with neurodivergent patients reported higher comfort scores than those without. No differences were observed across pairwise comparisons for ethnic groups for any domain.

**Table 2:** Pre-training survey responses (ANOVA results).

	Mean Difference (SE)		
Analysis Variable	How Common?	How Knowledgeable?	How Comfortable?
Age	***F(3,1622)*** ***=*** ***7.04; p*** ***<*** ***0.0001***	***F(3,1628)*** ***=*** ***3.71; p*** ***=*** ***0.01***	***F(3,1629)*** ***=*** ***6.52; p*** ***=*** ***0.0002***
21–30 vs 31–40	0.19 (0.13); *p* = 0.141	−0.32 (0.14); *p* = 0.024	***−0.45 (0.15); p*** ***=*** ***0.004***
21–30 vs 41–50	***0.5 (0.18); p*** ***=*** ***0.006***	***−0.54 (0.2); p*** ***=*** ***0.006***	***−0.78 (0.22); p*** ***=*** ***0.0003***
21–30 vs over 50	***0.66 (0.17); p*** ***=*** ***0.0001***	−0.13 (0.19); *p* = 0.479	−0.33 (0.21); *p* = 0.116
31–40 vs 41–50	0.32 (0.21); *p* = 0.126	−0.22 (0.23); *p* = 0.325	−0.33 (0.25); *p* = 0.18
31–40 vs over 50	0.48 (0.2); *p* = 0.016	0.18 (0.22); *p* = 0.398	0.12 (0.24); *p* = 0.623
41–50 vs over 50	0.16 (0.24); *p* = 0.498	0.41 (0.26); *p* = 0.116	0.45 (0.28); *p* = 0.114
Gender	***F(1,1604)*** ***=*** ***8.14; p*** ***=*** ***0.001***	F(1,1609) = 3.56; *p* = 0.06	F(1,1610) = 0.17; *p* = 0.68
Male vs Female	***−0.64 (0.20); p*** ***=*** ***0.001***		
Ethnicity	F(4,1620) = 0.71; *p* = 0.58	***F(4,1626)*** ***=*** ***4.14; p*** ***=*** ***0.002***	F(4,1627) = 1.55; *p* = 0.19
Caucasian vs African American		0.34 (0.13); *p* = 0.012	
Caucasian vs Asian		0.82 (0.37); *p* = 0.028	
Caucasian vs Hispanic		0.97 (0.36); *p* = 0.008	
Caucasian vs Other		0.11 (0.38); *p* = 0.773	
African American vs Asian		0.48 (0.39); *p* = 0.215	
African American vs Hispanic		0.63 (0.38); *p* = 0.096	
African American vs Other		−0.23 (0.4); *p* = 0.56	
Asian vs Hispanic		0.15 (0.52); *p* = 0.776	
Asian vs Other		−0.71 (0.53); *p* = 0.176	
Hispanic vs Other		−0.86 (0.52); *p* = 0.098	
Title	F(5,1612) = 1.80; *p* = 0.11	***F(5,1618)*** ***=*** ***5.55; p*** ***<*** ***0.0001***	***F(5,1619)*** ***=*** ***8.85; p*** ***<*** ***0.0001***
RN vs CRNA		−0.45 (0.51); *p* = 0.386	−1.02 (0.56); *p* = 0.069
RN vs MD		***1.29 (0.39); p*** ***=*** ***0.001***	***2.33 (0.43); p*** ***<*** ***0.0001***
RN vs Technician		0.76 (0.31); *p* = 0.014	−0.47 (0.34); *p* = 0.165
RN vs PCT/CA		−0.12 (0.3); *p* = 0.699	−0.71 (0.33); *p* = 0.034
RN vs Other		−0.38 (0.14); *p* = 0.005	−0.29 (0.15); *p* = 0.048
CRNA vs MD		1.74 (0.64); *p* = 0.007	***3.35 (0.7); p*** ***<*** ***0.0001***
CRNA vs Technician		1.21 (0.6); *p* = 0.043	0.55 (0.65); *p* = 0.397
CRNA vs PCT/CA		0.33 (0.59); *p* = 0.579	0.32 (0.65); *p* = 0.623
CRNA vs Other		0.07 (0.53); *p* = 0.9	0.73 (0.57); *p* = 0.204
MD vs Technician		−0.53 (0.49); *p* = 0.278	***−2.8 (0.54); p*** ***<*** ***0.0001***
MD vs PCT/CA		−1.41 (0.49); *p* = 0.004	***−3.03 (0.53); p*** ***<*** ***0.0001***
MD vs Other		***−1.68 (0.41); p*** ***<*** ***0.0001***	***−2.62 (0.44); p*** ***<*** ***0.0001***
Technician vs PCT/CA		−0.88 (0.43); *p* = 0.04	−0.23 (0.47); *p* = 0.616
Technician vs Other		−***1.14 (0.33); p*** ***=*** ***0.001***	0.18 (0.36); *p* = 0.618
PCT/CA vs Other		−0.26 (0.32); *p* = 0.416	0.41 (0.35); *p* = 0.242
Experience	***F(3,1617)*** ***=*** ***4.71; p*** ***=*** ***0.003***	F(3,1622) = 2.05; *p* = 0.10	F(3,1623) = 2.29; *p* = 0.08
0–5 years vs 5–10 years	0.36 (0.15); *p* = 0.019		
0–5 years vs 10–20 years	***0.55 (0.18); p*** ***=*** ***0.003***		
0–5 years vs over 20 years	0.27 (0.2); *p* = 0.181		
5–10 years vs 10–20	0.19 (0.23); *p* = 0.418		
5–10 years vs over 20 years	−0.09 (0.24); *p* = 0.696		
10–20 years vs over 20 years	−0.28 (0.26); *p* = 0.286		
Experience with Neurodivergent Patients	***F(1,1585)*** ***=*** ***7.5; p*** ***=*** ***0.006***	***F(1,1591)*** ***=*** ***40.2; p*** ***<*** ***0.0001***	***F(1,1592)*** ***=*** ***10.9; p*** ***=*** ***0.001***
No vs Yes	***0.30 (0.11); p*** ***=*** ***0.006***	***−0.76 (0.12); p*** ***<*** ***0.0001***	***−0.44 (0.13); p*** ***=*** ***0.001***

RN, registered nurse; CRNA, Certified Registered Nurse Anesthetists; PCT, patient care technician; CA, clinical assistant. Significant results are in bold italics.

### Higher post-training survey scores were observed relative to pre-training scores across all domains

**Figure 1:**
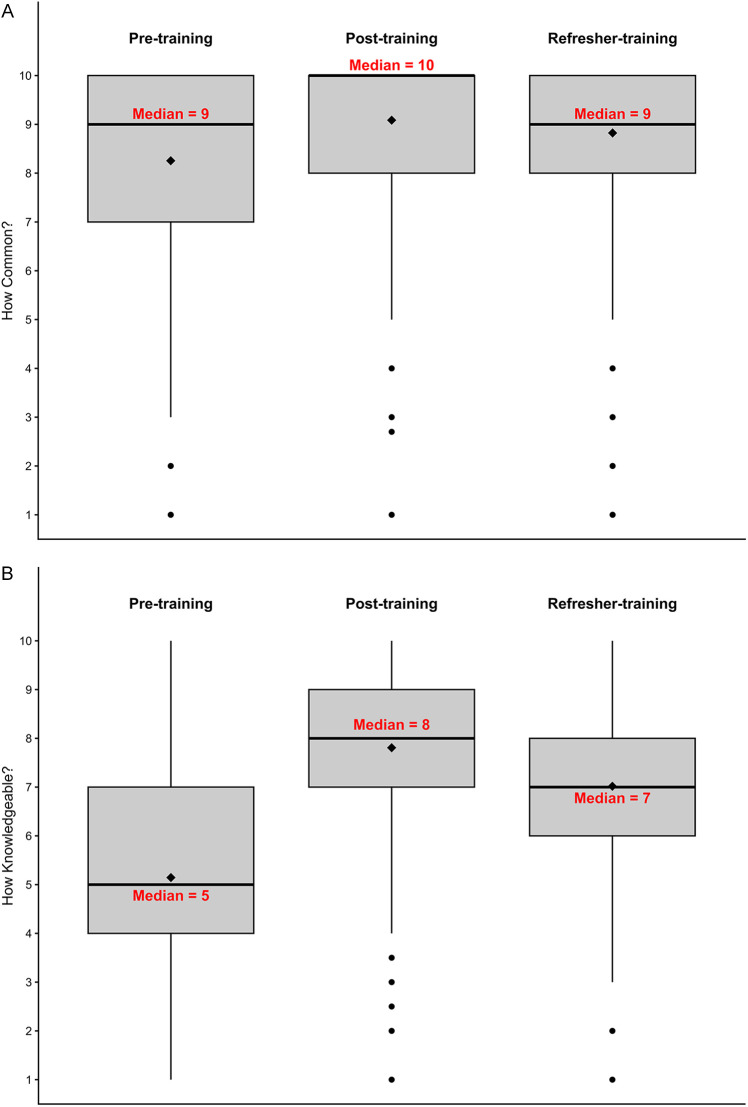

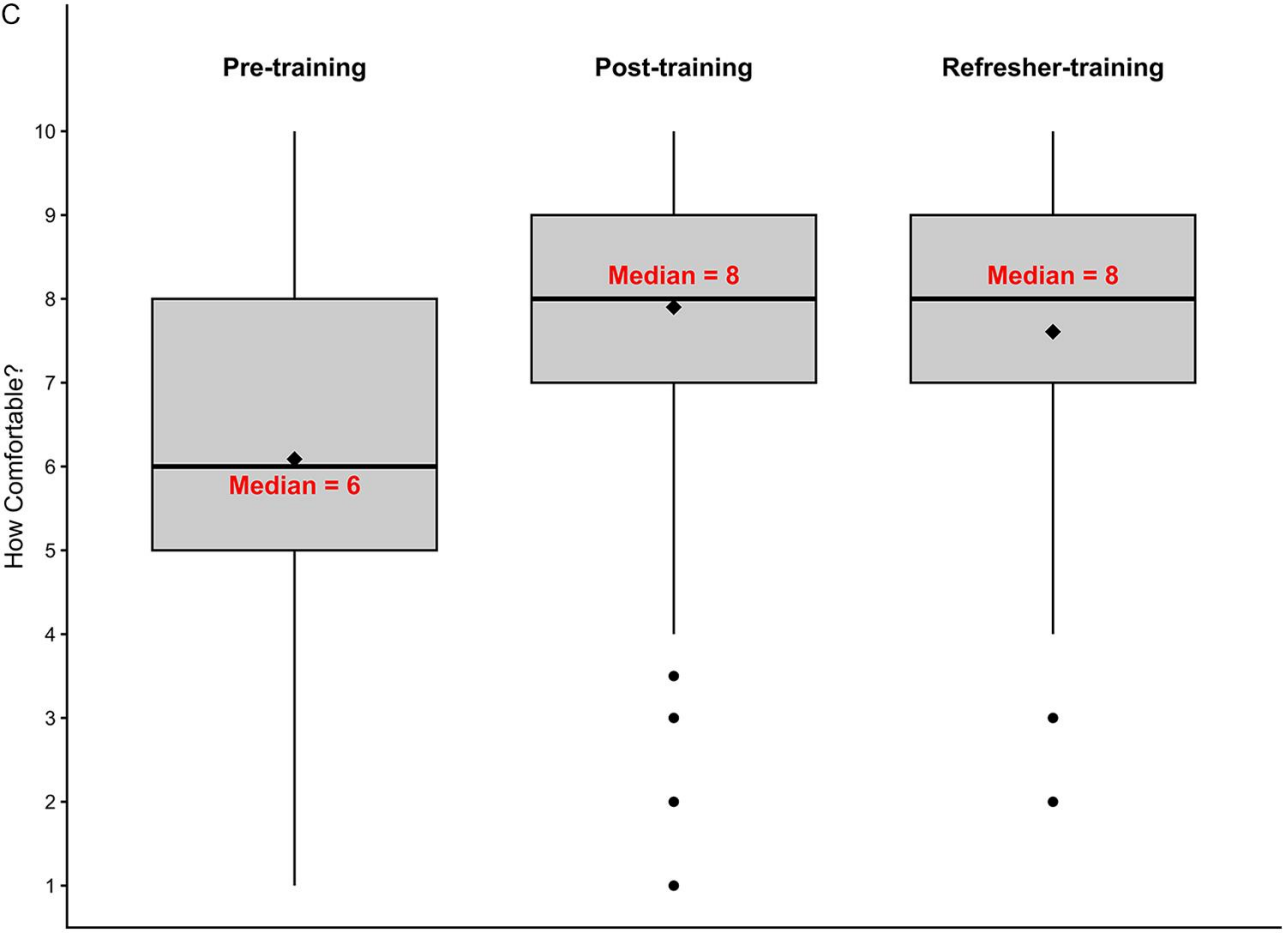
**(A)** Boxplots of scores to “how common?” Pre-. Post- and with Refresher Training. The box is bordered by the 25% percentile (Q1) and the 75% percentile (Q3) Inside the box are the median (line) and mean (diamond). The length of the line outside the box extends to 1.5* (Q3-Q2). Dots beyond the lines are considered outliers. **(B)** Boxplots of Scores to “How Knowledgeable?” Pre-. Post- and with Refresher Training. The box is bordered by the 25% percentile (Q1) and the 75% percentile (Q3) Inside the box are the median (line) and mean (diamond). The length of the line outside the box extends to 1.5* (Q3-Q2). Dots beyond the lines are considered outlier. **(C)** Boxplots of Scores to “How Comfortable?” Pre-. Post- and with Refresher Training. The box is bordered by the 25% percentile (Q1) and the 75% percentile (Q3) Inside the box are the median (line) and mean (diamond). The length of the line outside the box extends to 1.5* (Q3-Q2). Dots beyond the lines are considered outlier.

Figures 1A–C display survey response distributions across the pre-training, post-training, and follow-up survey periods. In the pre-training survey (*n* = 1639), participant response counts were 1,627 for how common, 1,633 for how knowledgeable, and 1,634 for how comfortable. The corresponding mean scores (±SD) were 8.25 ± 1.88, 5.14 ± 2.07, and 6.09 ± 2.27, respectively. In the post-training survey (*n* = 1630), response counts were 1,625, 1,626, and 1,624 for how common, how knowledgeable, and comfortable, respectively. A higher mean (±SD) score was seen in all domains (9.09 ± 1.35, 7.81 ± 1.45, and 7.90 ± 1.55, respectively). The mean scores of the post-training responses to how common relative to pre-training mean score was higher by 0.83 (95% CI: 0.72 to 0.94). The mean score of the post-training responses to how knowledgeable relative to the pre-training mean score was also higher by 2.66 (95% CI: 2.54 to 2.79). The mean score of the post-training responses to how comfortable relative to the pre-training mean score was higher by 1.81 (95% CI: 1.68 to 1.95). Using conventional benchmarks for Cohen's d (≈0.2 small, ≈0.5 moderate, ≥0.8 large), the standardized effect sizes indicate a moderate effect on perceived commonality (d = 0.51) and large effects on both knowledge (d = 1.49) and comfort (d = 0.93).

### Higher post-training quiz scores were observed relative to pre-training scores

Participants also completed an 11-item quiz assessing sensory related knowledge before and after training. Pre-training scores revealed a mean of 8.42 (SD 1.79) with the 50th and 75th percentiles at 9.0 and 10.0, respectively. Post training scores revealed a mean of 9.42 (SD 1.53) with 50th and 75th percentiles of 10.0 and 11.0, respectively. The difference in means between pre- and post-training quiz responses was 1.0.

### Higher post training follow-up survey scores were observed between 12 to >24 months relative to pre-training scores

A refresher training was conducted at 12 to >24 months after the initial training course. During the refresher training, a survey was administered (*n* = 223 at 12 month, *n* = 91 at 24 months, *n* = 40 at >24 months- these included participants who missed their refresher training, and took it after 24 months, *n* = 99 did not provide a response). Mean scores (±SD) among 453 participants were 8.80 ± 1.50, 7.00 ± 1.64, and 7.60 ± 1.73 for how common, how knowledgeable, and how comfortable, respectively. The scores remained higher than pre-training scores across all 3 domains. The mean score of the refresher training responses to how common relative to pre-training mean score was higher by 0.57 (95% CI: 0.73 to 0.41). The mean score of the refresher training responses to how knowledgeable relative to pre-training mean score was higher by 1.87 (95% CI: 2.05 to 1.69). The mean score of the refresher training responses to how comfortable relative to pre-training mean score was higher by 1.52 (95% CI: 1.71 to 1.33). The corresponding Cohen's effect sizes are 0.32, 0.95, and 0.70 for how common, how knowledgeable, and how comfortable, respectively.

### Most beneficial sensory intervention

**Figure 2:**
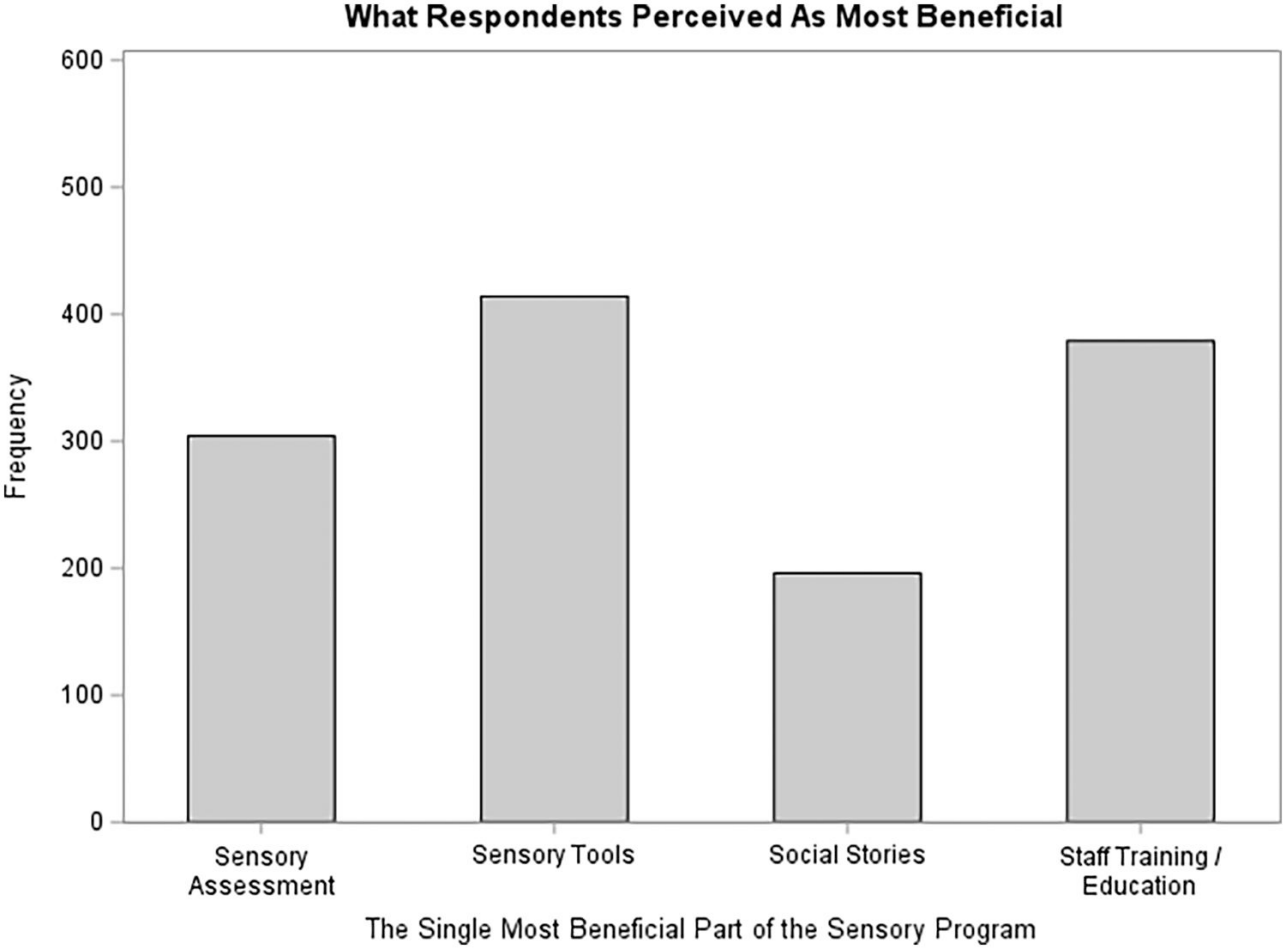
Frequency of respondents choosing most beneficial part of sensory program*. *Since more than one answer can be selected, the sum percentages can exceed 100%.

Sensory tools were identified as the most beneficial singular intervention (selected by 414 out of 479 participants, 86.4%) followed by sensory training as the next most beneficial intervention (selected by 379 out of 479 participants, 79.1%), Figure 2. Of note, participants were able to select more than one option.

## Discussion

Sensory processing challenges can be defined as difficulties in detecting, interpreting, and/or responding to exterior stimuli (auditory, visual, touch, taste, smell), interior stimuli (vestibular, proprioception, interoception) or combinations thereof (7). Children with sensory processing differences frequently experience challenges in communication and social interaction that intensify during acute illness (7–10). Prior research in emergency rooms and inpatient settings consistently supports the value of early identification, timely de-escalation, and implementation of sensory-informed interventions (including utilizing of sensory toolkits) in meaningfully improving care quality of neurodivergent patients (4, 11, 12).

Yet, healthcare staff often feel unequipped to support patients with sensory processing differences (13). This gap stems from insufficient training and awareness, as well as lack of sensory inclusive care resources that can lead to frustration and burnout in staff. Burnout, in turn, has been commonly linked to decreased job satisfaction and high turnover. Together, these factors are known to directly impact patient outcomes and the overall care experience (14).

This quality improvement initiative identified higher survey scores in provider-reported knowledge, comfort, and preparedness related to sensory-informed care following implementation of a structured sensory training within a pediatric hospital setting. Increase in objective post-training test scores supported the increased perception of knowledge. The training emphasized proactive strategies, including early identification of sensory triggers, adaptive communication techniques (e.g., use of simple, clear and concrete language), de-escalation approaches, and purposeful use of sensory tools to enhance delivery of care.

Follow-up survey scores collected prior to refresher training remained higher than baseline responses, suggesting persistence of reported knowledge and comfort over time within the institutional setting.

Higher post-training knowledge and comfort scores observed following implementation of the sensory training initiative may reflect increased provider-reported preparedness and confidence related to sensory-informed care within the institutional setting. In contrast, the smaller observed differences in perceived commonality may reflect relatively high baseline awareness, which may be less likely to vary across survey periods than knowledge- and confidence-based measures.

The observed associations across commonality, knowledge, and comfort highlight differences in baseline perceptions and experiential learning among respondents. Higher commonality scores among younger participants and those with fewer years of experience may reflect a shift in contemporary training culture toward neurodiversity-affirming, sensory-informed frameworks. In contrast, respondents with prior experience taking care of neurodivergent patients reported lower perceived commonality, potentially reflecting a more nuanced understanding of the heterogeneity and complexity of sensory needs that differentiates these patients from routine clinical encounters. Knowledge scores varied by age and professional role, suggesting that training curricula and scope of clinical interaction may influence familiarity and fluency with sensory accessibility principles, which in themselves could also serve as potential confounders in our observations. Respondents with prior experience with neurodivergent populations had higher knowledge and comfort scores compared to those without, underscoring the importance of experiential exposure in developing cognitive understanding and practical confidence. Notably, ethnicity and total years of experience were not associated with knowledge or comfort, suggesting that these competencies are more strongly shaped by role-specific training and direct exposure rather than demographic characteristics or cumulative time in practice alone.

Providing medical staff with sensory processing training equips them with the knowledge and skills needed to feel more confident and comfortable in their roles. Empowered staff approach their work with greater engagement and compassion, making interactions with patients and families more meaningful (15). That shift matters clinically as compassionate, attuned care can improve clinical outcomes and help reduce burnout (16).

For patients with sensory differences and their families, healthcare experiences can be isolating, with many reporting that their needs are often misunderstood or overlooked (17). These experiences can create mistrust in the healthcare system, which may lead to negative consequences including missed follow-up appointments, delayed intervention, and possible avoidance of medical intervention altogether (18). Sensory inclusive care directly addresses this. By modifying environments and adapting care approaches to meet patients where they are, providers can begin to close the gap between what patients need and what they actually receive.

At the bedside, providers reported that the most beneficial component in taking care of their patients was the utilization of sensory tools, followed by the training itself then patient sensory assessment and use of social stories. Examples of sensory tools include noise cancelling headphones, weighted blankets, light noise and motion producing toys, fidget toys, and stress balls that are individually selected to match the needs of the patient. For instance, a patient with noise, or an auditory sensitivity will be given noise canceling headphones, while a patient seeking an auditory stimulation will be given a sound machine. When used appropriately, these tools help regulate sensory input and reduce the impact of sensory dysregulation (19, 20).

Sensory assessments are standardized questions that delineate a patient's known triggers, calming items or strategies, and communication preferences that outline their sensory profile in a way that is easily accessible to any provider. Social stories, meanwhile, are first-person narratives told in simple, concrete language that prepare a patient for an encounter or procedure before they experiencing it in person (21). For example, before an intravenous blood draw, a bedside nurse will describe each step to the patient and show those steps visually via the social storybook. Having a detailed understanding of procedures beforehand helps with transitions, decreases anxiety, and potentially mitigates sensory overload.

Together, our findings suggest that sensory training may enhance staff awareness of patients with sensory needs and support the use of strategies and tools to reduce sensory overload, which may in turn contribute to improved patient communication and cooperation. Given that patients with sensory processing challenges frequently present with medical comorbidities and are therefore at increased risk for acute illness requiring hospital admission, enhancing healthcare personnel preparedness and reducing environmental barriers are critical steps toward improving outcomes for this vulnerable population. A multifaceted approach is essential for proactive sensory-informed care.

There were limitations identified in this study. First, because this project utilized anonymous unmatched surveys collected as part of a QI initiative, the findings are descriptive observations within a single institutional setting rather than evidence of causal intervention effects. Differences observed across the first and refresher training period may have been influenced by additional institutional, educational, or temporal factors that were not controlled within the project design. Additionally, voluntary participation in the survey may have attracted a subgroup of medical providers already interested in this field of research, showing selection bias, although this is unlikely as all providers who were in the hospital unit receiving the training as part of the hospital wide QI project took the survey. Outcomes were based on self-reported measures of knowledge, comfort, and perception of care, which introduces the possibility of response or recall bias. While there is also susceptible to social desirability bias, this is minimized as the responses were collected anonymously in an aggregate fashion. Importantly, these measures captured provider-perceived readiness and confidence, which are critical drivers of clinical behavior and implementation of inclusive care practices. Second, the study was conducted at a single tertiary pediatric hospital, which may limit generalizability to other institutional or resource settings and geographic regions; but that constraint came with meaningful trade-offs: standardized training delivery, and consistent institutional context, all of which strengthen internal validity and feasibility for broader dissemination. Third, participants were predominantly early-career, Caucasian registered nurses, which may represent regional demographic and therefore limit representativeness and application to a more diverse healthcare workforce. This group however reflects the frontline workforce who are engaged in bedside pediatric care, where sensory-focused interventions are likely to have the greatest immediate impact. Pre- and post-training responses were additionally unmatched and treated as independent responses, limiting individual-level comparisons with no objective measures of behavioral change. Patient-level outcomes were also not directly assessed. The follow-up survey group had a notably lower sample size, which reduces the reliability of the retention findings demonstrated. The lack of control group limits our ability to rule out secular trends or external factors contributing to observed improvements. Finally, the baseline quiz scores were high, suggesting a ceiling effect that likely limited the observable magnitude of improvement and therefore made the estimates conservative. However, the purpose of training is to achieve near-maximal performance; and since achieving the ceiling is the goal, this naturally becomes the bottom line.

Future research should focus on evaluating the impact of sensory-informed training on objective patient-level outcomes, including length of stay, procedural success, need for sedation, and patient and family satisfaction. Multicenter studies across diverse hospital settings are needed to assess generalizability and scalability of this intervention. Additionally, studies incorporating matched longitudinal data could better characterize individual-level knowledge retention and behavioral change over time. Exploring integration of sensory-informed care into standard clinical workflows, electronic health records, and institutional policies may further enhance sustainability. Finally, expanding research to include cost-effectiveness analyses and the impact on healthcare provider burnout and workforce retention would provide a more comprehensive understanding of the broader system-level benefits of sensory-inclusive care.

## Conclusion

Higher survey scores were observed in provider-reported knowledge, comfort, and preparedness related to caring for neurodivergent patients after implementation of a structured sensory training initiative within a pediatric hospital. These findings support the feasibility and perceived educational value of sensory-informed training and tools within clinical care environments.

## Data Availability

The original contributions presented in the study are included in the article/Supplementary Material, further inquiries can be directed to the corresponding author.

## Appendix 1: Sensory Assessment (correct answer highlighted in bold)

1. Sensory processing is the method that the nervous system uses to recognize, organize and make sense of incoming sensory input to create an adaptive response.

A. **True** B. False

2. Sensory sensitivity is not commonly encountered in the pediatric population.

A. True B. **False**

3. Which of the following are our human senses?
  A. Hearing and vision
  B. Taste and smell
  C. Vestibular and proprioception
  D. A and B
  E. **All the above**
4. Who are some of the patients who might have sensory sensitivity:
  A. Autistic individuals
  B. Down's Syndrome
  C. Blind and deaf patients
  D. Premies
  E. Fetal alcohol syndrome
  F. A, B and C only
  G. A and B only
  H. **All the above**
5. If a patient is holding his/her ears frequently or becomes distressed by a loud noise, the patient is demonstrating a sensitivity in what sensory processing system?
  A. Vestibular
  B. **Auditory**
  C. Visual
  D. Anxiety
  E. Proprioception
6. The following are signs of tactile dysfunction except:
  A. Fearful to touch
  B. Avoidance of hugs
  C. **Covers ears when sounds are too loud**
  D. Overacting to minor cuts, scrapes, or bug bites
  E. Withdraws hands when touched
7. Which of the following is not considered a sensory processing system?
  A. Proprioceptive
  B. Auditory
  C. Tactile
  D. **Anxiety**
  E. Vision
8. When a patient is experiencing proprioceptive dysfunction which of the following modifications would be most beneficial?
  A. Noise cancelling headphone
  B. **Weighted lap pad**
  C. Rice or bean bucket
  D. Puzzles
  E. Sunglasses
9. Some of the social impairments that patients with sensory sensitivity might exhibit include all of the following except:
  A. Difficulty communicating with others
  B. Difficulty in reciprocating
  C. Lack of interest
  D. Difficulty with adjusting to social expectation
  E. **Difficulty with mobility**
10. When speaking to an individual who is experiencing sensory over-stimulation, you should:
  A. Use a soft, calm voice
  B. Use a visual aid
  C. Try to reason with them
  D. Speak louder to get his or her attention
  E. **A and B**
  F. A, B and C
  G. All the above
11. When encountering a patient who is having a meltdown, you should do the following except:
  A. Keep the non-essential personnel away
  B. Go to the individual and offer help immediately
  C. Stand back to assess the situation
  D. Offer awareness of resources available
  E. Immediately touch the individual to provide comfort
  F. **B and E**
